# Implementing integrated care clinics for HIV-infection, diabetes and hypertension in Uganda (INTE-AFRICA): process evaluation of a cluster randomised controlled trial

**DOI:** 10.1186/s12913-023-09534-0

**Published:** 2023-06-02

**Authors:** Marie Claire Van Hout, Flavia Zalwango, Mathias Akugizibwe, Moreen Namulundu Chaka, Josephine Birungi, Joseph Okebe, Shabbar Jaffar, Max Bachmann, Jamie Murdoch

**Affiliations:** 1 Public Health Institute, Liverpool John Moores University, L32ET Liverpool, UK; 2 MRC/UVRI & LSHTM Research Unit, MRC/UVRI & LSHTM, Entebbe, Uganda; 3 University College London, London, UK; 4 University of East Anglia, Norwich, UK; 5 Kings College London, London, UK

**Keywords:** HIV, Non-communicable disease, Diabetes, Hypertension, Integrated care, Uganda

## Abstract

**Background:**

Sub-Saharan Africa is experiencing a dual burden of chronic human immunodeficiency virus and non-communicable diseases. A pragmatic parallel arm cluster randomised trial (INTE-AFRICA) scaled up *‘one-stop’* integrated care clinics for HIV-infection, diabetes and hypertension at selected facilities in Uganda. These clinics operated integrated health education and concurrent management of HIV, hypertension and diabetes. A process evaluation (PE) aimed to explore the experiences, attitudes and practices of a wide variety of stakeholders during implementation and to develop an understanding of the impact of broader structural and contextual factors on the process of service integration.

**Methods:**

The PE was conducted in one integrated care clinic, and consisted of 48 in-depth interviews with stakeholders (patients, healthcare providers, policy-makers, international organisation, and clinical researchers); three focus group discussions with community leaders and members (n = 15); and 8 h of clinic-based observation. An inductive analytical approach collected and analysed the data using the Empirical Phenomenological Psychological five-step method. Bronfenbrenner’s ecological framework was subsequently used to conceptualise integrated care across multiple contextual levels (macro, meso, micro).

**Results:**

Four main themes emerged; Implementing the integrated care model within healthcare facilities enhances detection of NCDs and comprehensive co-morbid care; Challenges of NCD drug supply chains; HIV stigma reduction over time, and Health education talks as a mechanism for change. Positive aspects of integrated care centred on the avoidance of duplication of care processes; increased capacity for screening, diagnosis and treatment of previously undiagnosed comorbid conditions; and broadening of skills of health workers to manage multiple conditions. Patients were motivated to continue receiving integrated care, despite frequent NCD drug stock-outs; and development of peer initiatives to purchase NCD drugs. Initial concerns about potential disruption of HIV care were overcome, leading to staff motivation to continue delivering integrated care.

**Conclusions:**

Implementing integrated care has the potential to sustainably reduce duplication of services, improve retention in care and treatment adherence for co/multi-morbid patients, encourage knowledge-sharing between patients and providers, and reduce HIV stigma.

**Trial registration number:**

ISRCTN43896688.

## Background

The global burden of non-communicable diseases (NCDs) continues to increase, with an estimated 41 million deaths annually, equivalent to 71% of all deaths globally (WHO, 2021). Cardiovascular disease accounts for the majority of NCD-related deaths (estimated 17.9 million per year globally), followed by cancers (9.3 million), respiratory diseases (4.1 million), and diabetes (1.5 million) [1]. Hypertension and diabetes are chronic long-term NCD conditions which represent a growing challenge to healthcare systems worldwide [2, 3]. These two conditions cause the most NCD-related mortality and morbidity, and disproportionately affect people living in low- and middle-income countries, where 85% of NCD-related deaths occur in people aged 30–69 years, and where women are disproportionately affected by the triple burden of NCDs, reproductive and maternal health conditions, and human immunodeficiency virus (HIV) [4–6].

In sub-Saharan Africa, HIV remains a leading cause of premature adult mortality, occurring alongside the increasing dual burden of NCDs [7–10] Increased urbanisation in the region, poor lifestyle (inadequate nutrition and sedentary behaviours), and poverty affects HIV, diabetes and hypertension rates, and related multi-morbidity and chronic ill-health [11–14]. Patient numbers in regular HIV care is increasing [15]. Hypertension represents the single commonest risk factor for death, with 78% of adults over 55 years living with hypertension, and with onset of both diabetes and hypertension occurring at a younger age [16]. Diabetes is also increasing, with 28 million adults living with the condition, and diabetes prevalence in the region is anticipated to double between 2010 and 2030 [17, 18]. NCDs are the leading cause of death and disability-adjusted life years for women older than 50 years [6, 16, 19–21]. Vulnerable and poor populations are especially impacted, and in particular, people living with HIV (PLHIV) [22, 23].

HIV vertical care in sub-Saharan Africa has allowed health services to focus resources on one condition and this, combined with differentiated models of care which allow easy access to medicines for PLHIV, has led to good outcomes for PLHIV. Management of diabetes and hypertension among PLHIV however remains a major health system challenge in the region [24], requiring lifelong care and support [25]. Patients are increasingly presenting with multi-morbidity with HIV, diabetes or hypertension, and earlier age of onset of diabetes and hypertension [12, 13, 26, 27]. Efforts to coordinate NCD care programmes alongside vertical HIV services continue to be developed and scaled up [27–33]. Integrated care defined as ‘*the coordination, colocation or simultaneous delivery of communicable and non-communicable services to patients who need it, when they need it*’ [34] is increasingly considered and implemented in various sub-Saharan African settings [14, 35–48]. To support and inform implementation and scale-up in various settings (hospital, primary care, community), large-scale trials with context-specific clinical, cost effectiveness, and process outcomes data are required [14, 43, 49, 50]. Evidence to date in sub-Saharan Africa remains largely confined to small-scale feasibility studies [14, 36, 51–55]. The potential impact of moving away from vertical approaches on HIV care also remains unknown.

We report here from Uganda which is experiencing a growing burden of NCDs, especially diabetes and hypertension across all socioeconomic strata (Table 1) [56].

**Table 1:** Uganda Profile

Income levels	Low income level (GNI per capita income of $840^1^)
Population size	49, 332,360 ^2^
HIV prevalence	6.2% ^3^
Diabetes prevalence	4.5% ^4^
Hypertension prevalence	31.5% ^5^
Doctors density/100 000 population	0.1((2015)^6^.

1.World Bank (2022). Global income classification issued on July 1, 2022. Available at https://www.worldbank.org/en/news/factsheet/2022/07/07/world-bank-country-classifications-by-income-level-uganda#

2. United Nations (2022). Worldometer. Available at www.worldometers.info/world-population/uganda-population/

3. The Uganda Population-Based HIV Impact Assessment (UPHIA) (2016–2017) (2018). Available at 3430•PHIA-Uganda-SS_NEW.v14.pdf (columbia.edu)

4. International Diabetes Federation (2021). Diabetes Atlas. Available at https://data.worldbank.org/indicator/SH.STA.DIAB.ZS?locations=UG

5. Lunyera J, Kirenga B, Stanifer JW, Kasozi S, van der Molen T, Katagira W, et al. (2018) Geographic differences in the prevalence of hypertension in Uganda: Results of a national epidemiological study. PLoS ONE 13(8): e0201001. Available at 10.1371/journal. pone.0201001

6. World Health Organization (2022). Global Health Workforce Statistics, OECD, supplemented by country data. Available at

https://data.worldbank.org/indicator/SH.MED.PHYS.ZS?locations=UG

**Fig. 1:**
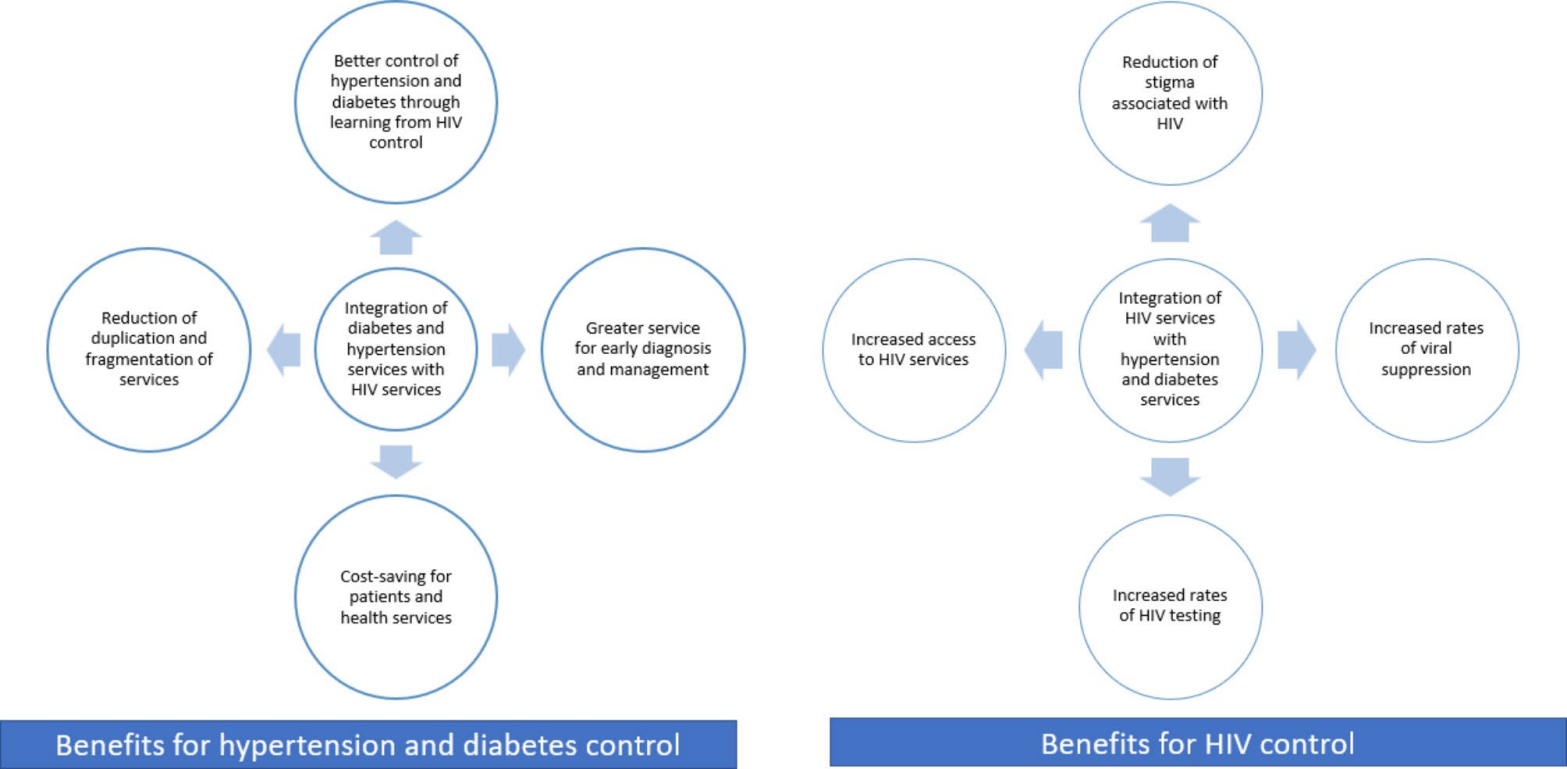
Expected benefits of integrated HIV, hypertension and diabetes care

Integrated HIV and NCD care was initially piloted by our team in a feasibility single-arm intervention study (2018–2020) in selected facilities in central Uganda (MOCCA study) [14, 36, 57]. Clinical and evaluation findings of this pilot study were encouraging. MOCCA was followed by a large-scale pragmatic parallel arm cluster randomised trial (INTE-AFRICA) which is a European Commission-funded project that aimed to test the efficacy of integrating diabetes and hypertension services alone, or together with HIV-infection services [49]. The intended outcomes of INTE-AFRICA were the reorganisation of clinics and staff to implement the integrated care model; less health service duplication; increased diagnosis of comorbid conditions; increased patient retention and adherence to treatment; an effective, quality and sustainably funded drug supply chain; and enhanced patient outcomes in terms of increased viral suppression, control of blood pressure and blood glucose (Fig. 1).

We report here on the outcomes of a process evaluation (PE) of the INTE-AFRICA trial which aimed to explore the experiences, attitudes and practices of a wide variety of stakeholders during implementation and to develop an understanding of the impact of broader social, structural and contextual factors on the process of service integration.

## Methods

The PE of the INTE-AFRICA trial aimed to garner an understanding of multi-stakeholder and multi-level perspectives of the social, behavioural, structural, contextual and cultural factors impacting on the process of implementation. These included individual and community health risks, protective behaviours and health responses within the broader social, community and political context (i.e., government resources and barriers to sustaining integration). The design was based on Medical Research Council guidance for evaluating complex interventions [58], and the PE was conducted in tandem with the collection of clinical outcomes (blood glucose, blood pressure, viral suppression, patient adherence) and health economic data (costs of integrated care delivery, and cost-effectiveness (compared to current standard care) during implementation of the trial.

### The INTE-AFRICA trial

Health facilities were stratified based on their infrastructure (district hospitals, health centres, not for profit health facilities) in the Kampala region of Uganda. The inclusion criteria of sites for scaled up integrated care were; lower level health facilities providing dedicated care for diabetes and hypertension, and HIV in separate clinics located some distance apart so that patients could not easily attend multiple clinics in one area. Other clinic considerations included patient numbers, drug supply management and access for research teams. Large regional and national referral hospitals were excluded. 17 urban, peri-urban and rural health facilities offering primary care services were selected based on inclusion criteria, previous experience with the MOCCA pilot study, and consultations between the research team and policymakers at the Ministry of Health, Uganda.

The 17 sites were subsequently randomised to provide integrated services for HIV-infection, diabetes and hypertension (integrated health education and concurrent management) or to continue with routine separate, vertical services. Clinics in the integrated arm operated a ‘*one stop’* service where participants, irrespective of their conditions, received health education on all three conditions in a shared waiting area, were managed by the same clinical team of doctors, clinical officers, nurses, and counsellors etc.), used a similar medical records format for all conditions, accessed medicines from a single drug dispensing point and accessed laboratory services from the same place. Cohorts of approximately 220 patients (110 living with HIV and 110 with diabetes and/or hypertension) were enrolled in each site and followed up for 12 months. The co-primary endpoints were retention in care in participants with diabetes and/or hypertension and, viral load suppression in PLHIV. Clinic services in both study arms were delivered by regular health workers. Research data were collected by research nurses from participants after routine clinical consultations or from their medical records. Patients who declined to participate in the research and trial participants in control arm clinics continued to receive standard vertical health care delivery. A total of 3605 participants were enrolled: 1918 living with HIV and 1687 with diabetes and/or hypertension.

### The PE theoretical Framework

Bronfenbrenner’s ecological model of behaviour [59–61] was chosen as a theoretical framework to conceptualise the introduction of integrated care as an event occurring within complex social systems operating across multiple contextual levels (e.g. macro, meso and micro health system contexts), and evaluate the delivery of integrated care and its causal assumptions. The framework provided an organising structure for investigating how the wider context of a complex health system (in this instance primary healthcare services) shaped the implementation of the health intervention (integrated care) on the ground. It is widely used both to understand the social determinants of health as well as for evaluating implementation [59–61]. A particular advantage of the Bronfenbrenner model over other frameworks is that it yields an understanding of social determinants of health as well as evaluation implementation; and overall facilitates an understanding of the interaction between the health system and community-contexts, for example, how ***macro*** contextual features (e.g. policy, HIV stigma discourse) are operationalised at a ***meso***-level (e.g. staff training, protocols, where patients are located and flows through clinics) and invoked at a ***micro-***level (e.g. circumstances that facilitate disclosure of condition, information sharing between patients).

### Implementation of the PE

The PE was conducted within one primary healthcare facility (formerly a HIV clinic providing anti-retro-viral treatment or ART) in the intervention arm. This site was selected for several reasons; it was sub-urban and served the largest patient population with HIV, diabetes and hypertension in the 17 sites, had a record of consistent drug supply for HIV and NCD drugs, and was within the restricted travel zone for the research team during COVID-19 restrictions.

Data collection consisted of 48 in-depth interviews with stakeholders (patient, healthcare provider, policy-maker, international organisation and clinical researcher); three focus group discussions with community leaders and members (n = 15); and 8 h of clinic-based observation in one day (8am to 5pm). Interviews and focus group discussions lasted between 30 and 60 min. Professional stakeholders were purposively selected to get a breadth of perspectives across the respective roles in providing integrated care.

Adult patients over 18 years were selected based on various criteria; the participant had to have been in the trial for 12 months; patients of both genders with a single conditions, as well as patients with co and multi-morbidity were included. Only patients residing within the restricted travel zone during COVID-19 restrictions partook. Very sick patients who needed immediate medical attention were excluded. All efforts were made to ensure maximum variation in experience of integrated care and experience of living with different combinations of conditions. Recruitment and data collection continued until data saturation was achieved.

Qualitative data collection guides and observational checklists were designed based on scoping reviews and Delphi studies [18, 20] conducted by our team, and team consultations. The tools aimed to facilitate an in-depth understanding of stakeholder perceptions and experiences regarding if and how integration of HIV, diabetes and hypertension functioned to improve the key intended outcomes of the trial and the management of chronic conditions. They were pilot tested and contained various dimensions of interest; the practicalities of accessing, providing and sustaining integrated services in terms of human resources and medicines supply; stigma and equity of access; catchment area/populations; health literacy; quality of care; barriers to access, retention and medicines adherence; waiting room dynamics; record keeping; retention across multi/co-morbidities; changes in healthcare provider roles, attitudes and patient relationships; and staff training requirements.

### The empirical phenomenological psychological five-step method

The Empirical Phenomenological Psychological five-step method [62] was used to combine psychological, interpretative and idiographic components during data collection and analysis. Data management was supported by NVivo.14. Following transcription, and back translation where required, data collection and analysis of qualitative data and observational checklist notes was iterative, moving between testing of emerging theories, and looking for convergent and divergent perspectives of care. The EPP stepwise method (see process evaluation protocol [50]) as inductive analytical approach [61] was closely adhered to in order to yield an assessment of the INTE-AFRICA trial implementation, a detailed description of the processes, relationships, complex socio-cultural and gendered processes in receiving and providing integrated care, and contexts involved in the delivery of integrated care, aspects of community positioning and identification of factors attributing to success or failure of integrated care. The process was additionally strengthened by triangulation of data across methods and sources, by working iteratively between interviews and observations, and focusing on points of convergence and divergence across the data, we were able to extend and refine the development of themes. resulting in the emergence of four main themes emerged; *Implementing the integrated care model within healthcare facilities; Integrated care and drug supply chains; Integrated care functioning to reduce HIV stigma*, and *Health education talks as mechanism for change.*

The Bronfenbrenner framework was subsequently used to a tool for conceptualisation of the implementation of integrated care across the various pertinent contextual levels (***macro, meso*** and ***micro)*** and focusing on the impact of the intervention on the Ugandan health system, and on components of the health system when evaluating delivery of integrated care and its causal assumptions.

### Ethical considerations

The INTE-AFRICA trial received ethical clearance and approval from three research and ethics committees. These are; the Liverpool School of Tropical Medicine (UK), the AIDS Support Organisation (TASO) Research and Ethics Committee and Uganda National Council of Science and Technology (UNCST).). Full protocol [Trial registration number ISRCTN43896688] is available elsewhere [49]. There was no prior relationship between researchers and participants. The researchers took time to explain the study details to the participants before obtaining written consent. Confidentiality and privacy of participants were observed and all interviews were conducted in the absence of onlookers.

## Results

Data were collected from 63 participants (Table 2).

**Table 2:** Socio-demographic characteristics of study participants

Variable	Participant category							
	Patients	Health care providers	Community members	Community leaders	Policy makers	Clinical researchers	International Organisation	Total
**Age**								
25–29	0	4	5	0	1	0	0	10
30–34	2	1	2	0	0	0	0	5
35–39	4	4	1	2	1	0	0	12
40–44	5	1	1	0	2	1	0	10
45–49	8	0	1	1	1	1	0	12
50+	11	0	0	2	0	0	1	14
**Sex**								
Men	12	1	5	3	5	1	0	27
Women	18	9	5	2	0	1	1	36
**Education**								
No education	0	0	0	0	0	0	0	0
≤ Primary (P.7)	3	1	0	0	0	0	0	4
≤ Senior four (S.4)	21	3	4	2	0	0	0	30
≤ senior six (S.6)	4	1	3	2	0	0	0	10
Diploma	2	2	1	0	0	0	0	5
Degree	0	3	2	1	5	2	1	14
**Marital status**								
Married	18	4	6	3	5	2	1	39
Single	8	6	3	2	0	0	0	19
Widow/widower	4	0	1	0	0	0	0	5
**Family status**								
Has children	30	8	7	5	5	2	1	58
Has no children	0	2	3	0	0	0	0	5
**Disease condition**								
Had HIV alone,	8	-	-	-	-	-	-	8
Had HTN alone	6	-	-	-	-	-	-	6
Had DM alone	4	-	-	-	-	-	-	4
Had HIV and HTN	3	-	-	-	-	-	-	3
Had DM and HTN	6	-	-	-	-	-	-	6
Had HIV and DM	2	-	-	-	-	-	-	2
Had all conditions	1	-	-	-	-	-	-	1
Total	30	10	10	5	5	2	1	63

### Implementing the integrated care model within healthcare facilities enhances detection of NCDs and supports comprehensive co-morbid care

The perceived benefits of receiving care for two or three different conditions within the same visit was pervasive across our interview discussions with various stakeholders, including the patients themselves. Various changes in routinized practices were observed to enhance chronic disease management within the integrated care system. Many policymakers and healthcare providers were aware that by providing more comprehensive screening, treatment and care within an integrated care setting potentially facilitated opportunities for clinical staff to identify undiagnosed conditions.

*“The opportunity that we have is that if the patient comes in with any of these three diseases, we can use the opportunity to test for the other two and if found positive, still we can treat those diseases in the same clinic and the patient is assisted. So our opportunity is that we have trained doctors to treat all the three conditions and we also have the necessary facilities including the structures.”* (Healthcare provider, interview, Female)

In particular, new diagnoses for patients with HIV was reported:

*“The HIV positive patients have not been having blood pressure measurements. We have found patients with undiagnosed hypertension. Some with diabetes have also not known that they have HIV so that opportunity that is there has allowed the improved screening and diagnosis of those chronic conditions”* (Clinical researcher, interview, Female)

Professional stakeholders indicated that comprehensive screening is a key component of integration, helping to discover other underlying conditions, identify health risk behaviours and promote health awareness and health literacy in patients.

*“For every adult patient our recommendation and advice is that as long as an adult patient comes to the facility, give them an opportunity to screen for hypertension, give them a basic screening about risk factors for hypertension or cardiovascular diseases in general or assess their health status, assess their consumption behavior, their other risk behavior, physical activities, assess their exposure to stress level..”* (Policy maker, interview, Male)

*“Patients were able to have all their blood pressure and glucose measurements done. In this integrated clinic they were able to benefit, those who are HIV positive were able to know that maybe [they] have developed hypertension and diabetes, because they [got] that chance of being tested.”* (Nursing officer, interview, Female)

*“It was an opportunity for us to be able to monitor out patients especially those with HIV that were not suppressing their viral load, so we were not understanding why such cases were happening. But now we are having all the health care providers in the same clinic, it is an opportunity for us to discuss cases amongst ourselves, and we are able to weed out such cases. So I see the opportunity that all the healthcare providers for the three conditions are on board which is helpful for us to treat these three conditions well.”* (Counsellor, interview, Female)

Narratives from policymakers also centred on several distinct patient and healthcare provider benefits underpinned by optimal utilisation of human and financial resources, and reduced duplication of services and patient care. Notwithstanding the increased capacity for screening, diagnosis, and treatment of previously undiagnosed comorbid conditions; and the broadening of skills of health workers to manage multiple conditions, patients with multiple conditions with limited resources were able to avoid multiple appointments and visiting several clinics.

*“The prevalence of HIV has now stagnated to over 6% of the population but the prevalence of NCDs is on the rise. So there is a double burden of NCDs in that particular population requiring urgent attention. Rather to have them in the same chronic clinic and be attended to holistically.”* (Policymaker, interview, Male).

This notion of holistic care was reflected in views of integrated care as helping to clarify patients’ expectations of what care would be provided, and how the system operated inside the clinic.

*“The person will come knowing where exactly to go and get treatment. They will see one doctor and get treatment for all three diseases at once. I think that is the major positive. That the patients are no longer moving up and down from one clinic to another trying to access the services.”* (Health care provider, interview, Female)

Interviews with patients and focus groups with community members revealed how for patients with two or three conditions, the ability to be treated and receive medicine in one visit for multiple conditions had obvious benefits on the amount of time, money and physical effort required by patients to get help for their condition.

*“It would help if I had all the three conditions and I come from far and I need to use transport. If they are to tell me to come on Monday, then Tuesday, and then Wednesday [vertical care appointments for different conditions], I do not have that kind of money. So if they give me one day, and I get all my drugs for all conditions at the same place, then it will be advantageous to me.” (*Community member, focus group, Women)

Similarly, healthcare providers and policy makers considered integrated care as functioning to reduce the financial and time burden on patients, enabling patients to continue with care-giving and employment responsibilities.

*“It saves time for the patient, they get services under one roof by one provider so it saves time, it saves money…you get better treatment outcomes when you integrate compared to vertical services.”* (Policymaker, interview, Male)

However, despite the consensus around patient time and resource saving, many patients described how integrated care led to longer consultations (often the whole day) and therefore longer waiting times and congestion in waiting areas. In contrast, the clinicians observed that spending longer consultations with a patient saved time and resources in the long run due to less frequent patient appointments.

*“The only issue that I see is that we spend a lot of time here when we come. Because the moment you come here on a clinic day, you have killed the whole day.”* (50-Year old patient with HIV, interview, Male)

*“If I was seeing a patient for HIV and maybe I’m using 5 minutes, now because I’m also going to see their diabetes and hypertension, I’m most likely going to use more time. Although in the long run you don’t have to see them frequently but at that moment there more effort that is needed.”* (Policymaker, interview, Male)

The longer waiting period was a particular problem for diabetic patients who needed to fast for longer periods of time (to measure their fasting blood glucose levels). Healthcare providers and members of the clinical research team also remarked that this potentially incurred invalid fasting glucose results.

*“If you are early and reach here at around 7 am, you might find around 20 people so you might leave at around 2.00 or 3.00 pm. It might reach 2.00 pm and you have not eaten. You are waiting for them to draw blood from you before eating.”* (59-year-old patient with diabetes and hypertension, interview, Female)

*“They are also waiting for long periods of time …we found it mostly affects patients directly with diabetes because they are told not to eat anything before, they see the Doctor. So, the patient comes at 8:00am and by the time they see the Doctor at 2:00pm, the sugar levels have completely deteriorated.”* (Clinical researcher, interview, Female)

### Challenges of NCD drug supply chains

This theme describes substantive insights about integration in terms of system capacity to ensure continuous supply and access to medication across all three chronic conditions. HIV drugs were reported by all stakeholders to be consistently available and boosted treatment adherence, with some stable patients able to access their drug refills for up to three months.

*“We have many services that we offer for HIV, because we have all the HIV medicine, we give them medicine for one month, for two months, or even three. Those that have stabilized don’t need frequent monitoring.”* (Healthcare provider, interview, Female)

In contrast, patients and healthcare providers reported on the apparent challenges in ensuring an effective quality and sustainably funded supply chain for NCD drugs. Patients were prescribed NCD drugs and if not available in the clinic had to buy privately.

*“The reason why these NCDs were not adequately treated, managed together with HIV, were related to the medicines of the NCDs. Some have not been regularly available and you could find that patients were getting ART [ antiretroviral therapy] but were not getting medicine for hypertension and diabetes, the health workers prescribe and ask them to go and buy [privately]. Sometimes they do not have money and the medicines are expensive.”* (Policymaker, interview, Male)

To overcome these problems, patients were encouraged and supported to form groups (‘*Medicine Clubs’*) where they each made a small financial contribution to co-opt joint purchasing of NCD drugs at subsidized prices.

*“Nurse taught us about it [patient support groups] and we made a group and collected some little money. We had constituted a committee that whenever we come, we give in the money and they register us.”* (53-year old patient with hypertension, interview, Female)

During COVID-19, the healthcare providers and community members/leaders described how sustaining these patient support groups and co-op medicine clubs was challenging. Restrictions on movement and reduced incomes hindered the ability of patients to make financial contributions to secure subsidised NCD drugs, thereby affecting their adherence to care, and their clinical outcomes.

***“****We were disturbed by COVID because we were told to go back to the villages and could not put money together because we were saving every little we could get for food since we were not working. Food was the most important that time not medicine.”* (Community leader, mixed focus group)

### HIV stigma reduction over time

This theme describes how integrated care appeared to have a destigmatising effect for people with HIV. However, this took time during the implementation process, and the initial implementation of integrated care was observed by healthcare providers and community members/leaders to be met with some resistance from patients with hypertension and diabetes who were now co-located at the integrated care clinic and receiving care alongside PLHIV. Patients in particular who were concerned around community or peer level identification as having HIV began to see and experience the benefits of integrated care in terms of confidentiality across all conditions.

*“At first when we had started the integrated clinic, those without HIV first resisted to be managed at the integrated clinic, but with time they got used to it, they now no longer face any issues or stigma with it.”* (Senior clinical officer, interview, Male)

*“They should integrate. You will not know what disease they [patients] have gone to treat... …if they put diabetes, hypertension and HIV in one room[clinic].*.” (Community member, focus group, Women)

An important factor in mitigating fear of HIV stigma appeared to be that the integrated care clinic purposely ensured that no patient’s medical condition was revealed along the care process, from arriving at the clinic and waiting to be seen, being triaged and consulted. Healthcare providers and operating protocols also ensured that patients’ medical records for all conditions were stored in the same place and looked the same. These strategies to reduce stigma along with the small number of trusted healthcare providers at the integrated care clinic, was observed by healthcare providers, patients and community members/leaders to enable patients to retain privacy and agency over their health, and promote a strong therapeutic relationship with their treatment providers.

*“Knowing that you have incurable diseases is a social shock, that’s why some people prefer to be known by only a few health workers. If such a patient comes and doesn’t find her or his preferred health workers, some even go back home without the medication, so if we integrate such patients’ secrets will be catered for.”* (Representative of non-governmental organisation, interview, Female)

Clinic observations and patients described how this respectful clinical environment appeared to lead to many patients choosing to disclose their condition to other patients in the waiting area, resulting in knowledge sharing around health and the respective conditions, and helped to create a sense of an integrated care community.

*“We talk and discuss, you can tell your friend how the disease is treating you, another person will tell you how they are managing to treat it. Everyone talks about how the diseases affects them* (53-Year old patient with hypertension, interview, Female)

*“Patients would be seen and heard talking to each other and advising each other to feed on greens and bitter vegetables”* (Researcher fieldnotes, clinic observation)

Such sharing of experiences among patients was described by many stakeholders as an indirect positive outcome of integrated care, and helping to addressing some of the psychological challenges associated with living with chronic conditions.

*“When I had just been diagnosed with diabetes, I thought that I was going to die and leave my children very young, but slowly I have been able to talk to people and someone tells you that they have been with diabetes for 20 years. So that has strengthened me and I’m now okay I don’t have any worries of death.. I’m strong I have decided to keep taking my medicine to stay alive”* (56-Year old patient with diabetes, interview, Female)

### Health education talks as a mechanism for change

This theme describes how health awareness raising and reciprocal knowledge sharing between healthcare provider and patient, between patients, and in communities occurred during integrated care implementation. Patients with different conditions came to experience a sense of shared knowledge and experience was through the health education talks provided in the waiting areas at the integrated care clinic. Health workers (mainly nurses) spoke to patients about nutrition with most conversations centring on cautioning patients about unhealthy lifestyles and informing them about lifestyle modification (discouraged eating fatty foods, drinking alcohol, feeding on bitter foods, greens and against taking too much sugar). Other talks focused on issues regarding stress management, physical activity and adherence to medication. Patients received these talks while seated at the waiting area as a group, and some also received individually targeted talks while at the health workers’ desks.

*“When patients are in one clinic, they all receive same information and this gives them a chance to have similar understanding and again, if there is need to ask, they can ask amongst each other and get to know exactly what to do..”* (Policymaker, interview, Male)

Exposure to education about different health conditions during waiting times and the ability to discuss with fellow patients was reported as having various consequences for both healthcare providers and patients themselves. As well as patients with different conditions sharing their own knowledge with one another, the health education talks appeared to prime patients with questions for their subsequent one to one consultation with a clinician.

*“Like when we combine them, HIV, hypertension and diabetes, they get to know [about] diseases [other than] HIV, and they get to know how to [manage them]in future. I am talking about these people who have not yet got that disease like hypertension and diabetes.*” (Counsellor, interview, Female)

In addition, sharing knowledge about different conditions also cascaded across health workers, who described increased skills and confidence in treating different conditions.

*“I have learnt how to manage all patients, HIV s, diabetic and hypertensives. We become considerate to all of them.”* (Counsellor, interview, Female)

*“The staff (the health workers) who are managing HIV have gained confidence in managing NCDs and those who have managed NCDs have gained confidence in managing HIV. Patients can be managed comprehensively by one clinician.”* (Clinical researcher, interview, Female)

Policymakers took a broader perspective on the capacity-building of health care workers. Integrating care of HIV alongside diabetes and hypertension not only increased their knowledge across these different conditions but also informed the wider research and policy context into which such care was being provided, by leveraging a framework to align policy and practice on the management of NCDs.

*“The other opportunity that exists is the vast experience of knowledge especially that the low and middle income countries have, they have developed in research and increase in knowledge used in the HIV platform. And looking at that capacity, we have more health care workers leaning towards research, we have health care workers that have capacity in terms of policy using the HIV platform. It is an easy opportunity for us to leverage those opportunities and develop the rightful framework for management of NCDs.”* (Policymaker, interview, Male)

**Fig. 2:**
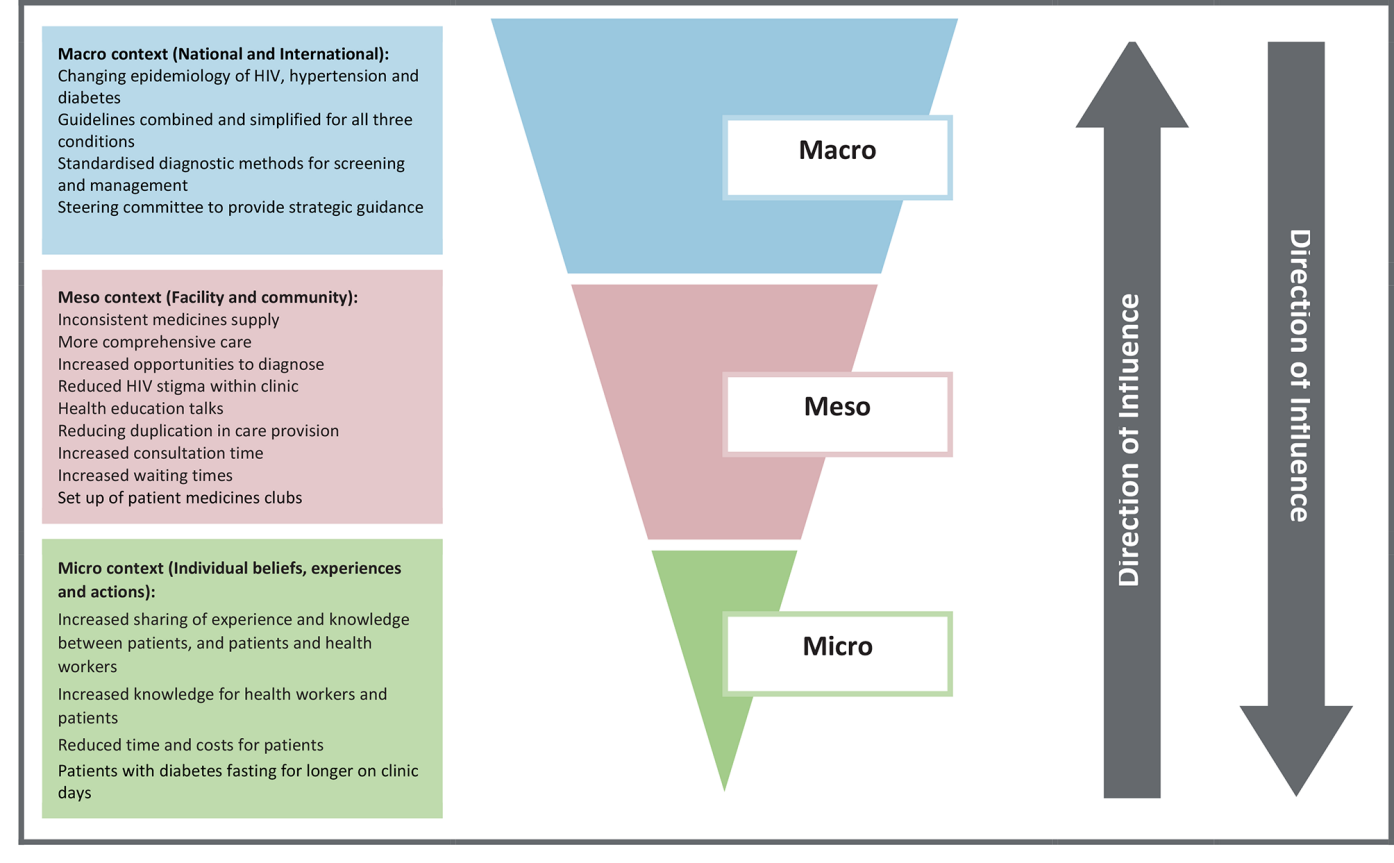
Contextual influences on INTE-AFRICA programme implementation cascade in Uganda

Using the Bronfenbrenner theoretical framework [59, 60] the four distinct themes suggest that strategic guidance (***macro-level***) to integrate care of HIV, diabetes and hypertension, operationalised by combining and simplifying care across the three conditions, opens up new opportunities for providing care within healthcare facilities (***meso-level***). Delivering health education talks to all patients, and a purposive strategy to anonymise patients’ conditions along the care pathway, facilitated a space for the increased sharing of knowledge that we observed between patients and providers within waiting areas and consultations (***micro-level***) (see Fig. 2). These insights represent directions for contextually relevant strategies of how integrated care for HIV and NCDs might function to sustainably improve retention in care and treatment adherence for co-morbid patients with HIV, and patients with different NCDs, not only within the specific site participating in this PE but on a much wider scale. Such strategies however need to be supported by consistent NCD drug supply either through well-resourced supply chains or via the support of patient medicines clubs.

## Discussion

By interpretation of multi-stakeholder navigation of integrated care, the PE supports greater in-depth multilevel understanding of integrated care in Uganda and helps avoid the *‘black box’* problem in interpreting trial results by improving understanding of the mechanisms that connect particular intervention components to particular outcomes [63]. Findings are largely in line with various studies in sub-Saharan Africa, and contribute to the growing evidence base on integrated management of HIV and NCDs in the region.

Generally, the PE demonstrated multi-stakeholder approval of integrated care for HIV, diabetes and hypertension in the Uganda setting due to the broad range of patient, healthcare provider, health system and community level benefits incurred. Despite resource constraints impacting on drug supply, key findings centre on several areas; high retention rates, acceptability and avoidance of duplication of care processes for people with multiple conditions; increased capacity for screening, diagnosis and treatment of previously undiagnosed comorbid conditions; broadening of skills of health workers to manage multiple conditions; reduction of HIV related stigma, development of peer level sharing of health information, informal groups (‘medicines clubs’) sharing health related information and purchasing of NCD drugs during clinic stockouts.

Challenges to multi-morbid chronic disease care centre on linkage and retention in care, access to medicines and adherence to treatment [43, 64]. A Delphi consensus study on best practice on integrating diabetes, hypertension and HIV care in Africa has identified as key components of optimal integrated care such as improved surveillance, strengthened drug procurement systems, availability of equipment and access to relevant blood tests, multi-condition health education and enhanced multi-morbidity continuity of care [20]. Elsewhere other assessments of patient and provider experiences of integrated NCD and HIV care in Sub-Saharan Africa (mostly in South Africa, Malawi, Uganda, Tanzania and Kenya) report on high patient demand, staff capacity and workload issues, long waiting times, NCD drug supply issues and HIV related stigma [20, 50, 57, 65–74]. Difficulties in having to pay for diabetes and hypertension medication (in contrast to HIV drugs) and transport, and choosing between food and medicine, are also reported in other integrated care evaluations [41–43, 57, 65, 73, 75]. Capacity issues were observed in response to high demand (also due to new diagnoses of previously undetected disease), leading to long waiting times for patients. Despite encountering and navigating similar challenges in delivery of integrated care (for instance infrastructure and human resource capacity, drug supply), the INTE-AFRICA trial was well received by health policymakers, healthcare providers in the clinic, the patients receiving care and in the community.

We speculate that the observed initial experiences of HIV related stigma attached to the clinic may have influenced patient experiences and journeys along the integrated care continuum. The process was dynamic over time, most notably with regard to the minimisation of HIV related stigma. Initial healthcare providers’ misgivings about risk of disrupting HIV care were overcome, leading to staff motivation to continue providing integrated care to their patients. It is encouraging to see that over time this HIV related stigma reduced, along with patient commitment to receiving treatment in a holistic, trusted and confidential environment. Managing HIV alongside any other chronic condition offers substantial opportunity to reduce stigma and can increase effective control of viral loads [67].

Finally, and perhaps most importantly, the PE revealed insights into indirect or unexpected consequences of integrated care, in terms of the developed *‘peer community irrespective of disease status’*, and the sense of mutual sharing of experience and health literacy among patients at the clinic, leading to opportunities for informal health education and peer support, sensitization around HIV stigma, and the co-operative approve to securing NCD drugs for everyone. Health workers and patients also reported being better educated about various clinical aspects of NCDs. Health education talks potentially facilitated patients asking targeted questions about their condition which might have improved blood pressure/blood glucose control. Integrated care in this sense via community awareness raising possibly promotes adherence to better diet, less salt, more activity, and hypertension drugs.

### Limitations

Limitations of the PE centre on the restriction of assessment to one large integrated care clinic, the relatively small sample size of participants, and the possibility for bias and socially desirable responses by patients and community participants.

## Conclusion

The NCD Alliance is calling for increased integrated NCD prevention, treatment and control within existing health and HIV programmes and services DS [6]. The PE interpreted multi-stakeholder navigation of integrated care in Kampala, Uganda and supports greater in-depth multi-level understanding of integrated care within the sub-Saharan African context. Implementing integrated care has the potential to sustainably reduce duplication of services, improve retention in care and treatment adherence for co/multi-morbid patients, encourage knowledge-sharing between patients and providers, and reduce HIV stigma. Robust NCD drug supply chains are imperative to support adherence to treatment and retention in care. Further implementation science research is warranted to garner greater understanding of patient experience and reported health outcomes, including a focus on their quality of life when experiencing multi-morbidity, and when accessing integrated care models in various contextual settings (hospital, primary care, community). Evaluations of integrated models of care in different contexts in sub-Saharan Africa can support context adaptation and a platform for the sharing of best practices, lessons learnt, health system reconfigurations and facility level standard operating procedures [24, 49, 50]. The growing evidence base in the region should not hold up domestic health system innovation and scale up of integrated care.

## Data Availability

Due to some data in the transcripts containing information attached to the health conditions of the patients that could not completely be anonymized, participants did not consent to having their data publicly availed. However, on request, data to the findings of this study can be accessed from MRC/UVRI and LSHTM Social Science server through authorization from the team lead investigator Professor Marie Claire Van Hout. M.C.VanHout@ljmu.ac.uk.

## Declarations

### Authors’ information (optional)

All authors hold PhDs, with exception of one researcher M.NC with a Bachelor’s degree, and three researchers F. Z, M. A, and B. J educated to Masters level. The team was comprised of both males and females.
